# The assessment of psychopathology among traumatized refugees: measurement invariance of the Harvard Trauma Questionnaire and the Hopkins Symptom Checklist-25 across five linguistic groups

**DOI:** 10.1080/20008198.2017.1321357

**Published:** 2017-06-09

**Authors:** Tim R. Wind, Niels van der Aa, Simone de la Rie, Jeroen Knipscheer

**Affiliations:** ^a^ Department of Research, Arq Psychotrauma Expert Group, Diemen, the Netherlands; ^b^ Department of Clinical Psychology, Utrecht University, Utrecht, the Netherlands

**Keywords:** Refugees, mental health, confirmatory factor analysis, validity

## Abstract

**Background**: Questionnaires are widely used to assess the mental health status of refugees, whereas their construct validity largely remains unexplored.

**Objective**: This study examined the construct validity of two widely-used instruments for the assessment of PTSD symptoms (Harvard Trauma Questionnaire [HTQ]; 16 items) and symptoms of anxiety and depression (Hopkins Symptom Check list-25 [HSCL-25]; 25 items) among Dutch and refugee patients with different linguistic backgrounds.

**Method**: We applied exploratory factor analyses and measurement invariance analyses to test construct validity.Participants (*n* =1 256) were divided into five linguistic groups defined by language family, including four non-western linguistic groups (Indo-Iranian [*n* = 262], Niger-Congo [*n* = 134], Semitic [*n* = 288], and South Slavic languages [*n* = 199]) and one western linguistic group (Germanic languages; Dutch [*n* = 373]).

**Results**: Exploratory factor analysis yielded a 3-factor structure of the HTQ and a 2-factor structure of the HSCL-25. Measurement invariance 20 analyses on the HTQ showed strong measurement invariance across the groups of refugee patients. However, Dutch patients reported milder symptom severity on most items of the HTQ. Measurement invariance analyses on the HSCL-25 (not conducted in Dutch patients) indicated partial strong measurement invariance across refugee patients.

**Conclusion**: We conclude that mental health constructs measured by the HTQ and the HSCL25 25 are to a large extent interpreted in a similar way by refugee patients. This indicates that these instruments can be applied in non-western refugee patient populations, and that local idioms of distress and inherent response patterns may not play a major role when applying the HTQ and the HSCL-25 in these populations. Yet, whereas meaningful comparisons of observed PTSD and depression scores between groups of refugee patients with different non30 western linguistic background are feasible, comparisons between patients with a western and non-western linguistic background, as well as comparisons of anxiety scores, are likely to be biased. Future studies need to establish whether the commonly used cut-off scores of both questionnaires apply for refugee patients with non-western linguistic backgrounds.

## 1. Introduction

On the borders of Europe the initial hope of the Arabic democratic uprisings in 2011 (the Arab Spring) has faded away and in some cases this political transformation has surmounted in protracted civil wars such as in Syria. The consequent influx of refugees in Europe (UNHCR, 2015) is currently dominating the news and the political debate. War experiences, persecution, hunger, loss of loved ones, a long and unsafe journey, and settlement in refugee camps all take their mental and physical toll (de Jong, Komproe, & Van Ommeren, 2003; Hassan, Ventevogel, Jefee-Bahloul, Barkil-Oteo, & Kirmayer, 2016). The resulting long- and short-term mental health and psychosocial consequences are many and varied, and a proportion of refugees seek health care for these mental health problems in their host country (de Jong, 2002; de Jong, Komproe, & Van Ommeren, 2003; Hassan et al., 2016).

To assess the impact of experiences among arriving refugees, tools such as mental health questionnaires are widely used (e.g. Buhman et al., 2014; Hollifield et al., 2013). At the individual level, assessment tools help clinicians to triage patients, target symptoms, and to assess treatment outcomes (Rasmussen, Verkuilen, Ho, & Fan, 2015). At the group level, assessments provide information about subpopulations that need treatment resources, therapeutic modalities that are more effective, and mental health information about patient populations in general (see Narrow, Rae, Robins, & Regier, 2002; Rasmussen et al., 2015).

In a previous editorial of this journal, Olff (2015) highlighted that the selection of the appropriate instruments to assess psychological complaints among refugees is not an easy task. Within this selection process, clinicians also need to bear in mind whether an instrument is valid across different linguistic groups (see Fodor, Pozen, Ntaganira, Sezibera, & Neugebauer, 2015). Health professionals are using assessment tools in linguistic groups for whom these instruments were not originally developed (Miller, Kulkarni, & Kushner, 2007; Rasmussen et al., 2015).

Questionnaires are used to measure a variety of complaints and emotions, such as depression, anxiety, and post-traumatic stress symptoms. Despite evidence of underlying universality in the experience of these mental health complaints, differences in the salience, manifestation, and expression of symptoms may be substantial across various cultures (e.g. Sweetland, Belkin, & Verdeli, 2014). In many non-western contexts the words ‘depression’ or ‘anxiety’ do not have direct equivalents. A local language may use a number of expressions, metaphors, proverbs, or emotion words to express a complaint or an emotion that is quite different from western jargon (de Jong, 2002; Kaiser et al., 2015). The way people label complaints or emotions are termed ‘idioms of distress’ (de Jong, 2002).

Differences in idioms of distress may simply refer to a different wording of the same mental health concept, but at worst these linguistic differences may reflect actual differences in mental health concepts (Miller et al., 2007; cf. Wind, Joshi, Kleber, & Komproe, 2014; Poortinga, 1975). In the latter scenario, the consequence could be that the covariance between the items of the questionnaire that refer to the latent mental health concept may be different across linguistic groups (see Dyer, Hanges, & Hall, 2005). Thus, differences in idioms of distress may ultimately undermine the validity of mental health questionnaires that were developed in western languages. We do not know which concept holds true until we examine the validity of mental health questionnaires among linguistic groups.

Building upon previous research on global mental health in this journal (Hall & Olff, 2016; Purgato & Olff, 2015), the aim of this study was to examine whether two widely used mental health questionnaires – Harvard Trauma Questionnaire (HTQ) and Hopkins Symptom Checklist-25 (HSCL-25) – assess the same mental health concepts across groups of refugees with different linguistic backgrounds. If the instruments measure the same mental health concepts across groups with different linguistic backgrounds, the mental health questionnaires are called ‘measurement invariant’ across these groups. If measurement invariance (MI) can be demonstrated, this implies that the items of the mental health questionnaires as well as the mental health concepts they are measuring are interpreted in the same way by individuals with different linguistic backgrounds (Horn & McArdle, 1992; Van De Schoot, Lugtig, & Hox, 2012). Only when MI holds for a mental health questionnaire, cross-group differences in scores on mental health constructs are meaningful (Meredith, 1993; Steenkamp & Baumgartner, 1998; Van De Schoot et al., 2012). Methodologically, scholars have examined MI of mental health questionnaires using confirmatory factor analyses (CFA; Charak et al., 2014; Contractor et al., 2015; Fodor et al., 2015; Schnyder et al., 2015). CFA is a widely-used technique for testing MI of mental health questionnaires.

The wealth of factor analytic research on mental health questionnaires has not been linguistically and culturally evenly distributed. Studies from non-Euro-American samples are exceedingly sparse. Fodor et al. (2015) examined the factor structure of PTSD within a group of Rwandan adults who experienced trauma during the 1994 genocide using the PTSD-Checklist – Civilian Version (PCL-C). Their results suggest that the latent structure of PTSD found in this sample was comparable to Euro-American samples. Charak et al. (2014) found that the Dysphoric Arousal Model of PTSD assessed by the PCL-C was the best model in an Indian sample, although the fit indices of all PTSD models were fairly similar, which underlines the cross-cultural validity of PTSD symptomatology. In a study by Contractor et al. (2015), the structural invariance of PTSD 5-factor model across Hispanic and Caucasian groups was supported.

Previously, Rasmussen and colleagues examined MI of the most widely used PTSD measure in refugee populations, the HTQ (Mollica et al., 1992). They showed that posttraumatic stress is conceptually comparable in a multinational and multilingual sample of asylum seekers from 81 countries of origin in 11 global regions, yet comparisons of mean and sum scores and of symptoms over time were not meaningful. These findings called into question the common practice of using standard cut-off scores on PTSD measures across culturally dissimilar refugee populations.

Thus, we examined MI of symptom severity of depression and anxiety as assessed by the HSCL-25 and of posttraumatic distress as assessed by the HTQ in a large sample of refugees across four non-western linguistic groups (Indo-Iranian languages, Niger-Congo languages, Semitic languages, and South Slavic languages) and one western linguistic group (Germanic language).

## 2. Method

### 2.1. Participants

Participants were Dutch and refugee patients referred for treatment at Foundation Centrum ’45, a specialized Dutch centre for treatment and diagnosis of complex psychotrauma (i.e. PTSD with comorbid disorders). In 2001, Foundation Centrum ’45 started to routinely monitor treatment outcomes by administering questionnaires to patients during treatment. For the present study, participants were included for whom intake data collected with the HTQ and/or the HSCL-25 were available. Because the HSCL-25 was only conducted to refugee but not to Dutch patients, no data collected with the HSCL-25 were available for Dutch patients.

Because refugees with a large variety of native languages participated, homogeneous groups were composed based on the language family to which the language of the refugee’s country of origin belongs (Katzner, 2002). Language family can be defined as a group of languages which are related because they descend from a common ancestor. Languages within the same family have observable shared characteristics that are not attributed to contact or borrowing. Data on the HTQ and/or the HSCL-25 were available for 1717 participants. A total of 1256 (73%) participants were divided into five main linguistic groups defined by language family (Katzner, 2002): Indo-Iranian languages (included: Iran, Afghanistan); Niger-Congo languages (included: Angola, Burkina Faso, Burundi, Cameroon, Cote d’Ivoire, Gambia, Guinea, Kenya, Liberia, Nigeria, (Democratic) Republic of Congo, Rwanda, Sierra Leone, Togo, Uganda); Semitic languages (included: Algeria, Egypt, Eritrea, Ethiopia, Iraq, Kuwait, Lebanon, Libya, Palestine, Syria); South Slavic languages (included: Bosnia and Herzegovina, Croatia, Macedonia, former Yugoslavia); and Germanic languages (included: The Netherlands). Sample sizes of the linguistic groups of the remaining 461 (27%) participants were too small (*N* = 1–31) to make a fair comparison between linguistic groups. Therefore, these participants were excluded from the analyses. In the upper part of Table 1, sample sizes and demographic characteristics for the total sample, as well as the five linguistic groups, are presented. Participants with a Germanic (30%) and Niger-Congo (11%) linguistic background constituted the largest and smallest subsample respectively. Sample sizes of the linguistic groups for the MI analysis of the HTQ ranged between 132 and 373, and between 123 and 257 for the MI analysis of the HSCL-25. Participants were mostly male (71%) and had a mean age of 43.3 years.

**Table 1.** Demographic characteristics and symptom severity with regard to PTSD, anxiety and depression for the total sample and the five linguistic groups.

	Total sample	Indo-Iranian	Niger-Congo	Semitic	South Slavic	Germanic
Total sample size	1256	262	134	288	199	373
Sample size for HTQ	1247	259	132	286	197	373
Sample size for HSCL-25	756	218	123	257	158	N.A.
Gender: *n* (%) males	896 (71%)	200 (76%)	77 (58%)	227 (79%)	131 (66%)	261 (70%)
Mean age (*SD*)	43.3 (11.0)	42.4 (11.0)	30.9 (7.9)	42.2 (9.6)	45.5 (9.0)	48.1 (10.0)
Mean PTSD symptom severity (*SD*)	2.9 (.6)	3.0 (.6)	3.0 (.6)	3.1 (.5)	3.1 (.5)	2.5 (.7)
*N* (%) symptomatic for PTSD	913 (74%)	207 (81%)	102 (78%)	252 (89%)	168 (86%)	184 (49%)
Mean (*SD*) symptom severity anxiety	2.9 (.7)^a^	2.8 (.7)	2.9 (.7)	3.0 (.6)	3.0 (.6)	N.A.
*N* (%) symptomatic for anxiety disorder	713 (94%)^a^	200 (92%)	117 (95%)	243 (95%)	151 (97%)	N.A.
Mean (*SD*) symptom severity depression	2.9 (.6)^a^	2.9 (.6)	3.0 (.6)	3.0 (.6)	2.9 (.6)	N.A.
*N* (%) symptomatic for depressive disorder	721 (96%)^a^	206 (96%)	117 (95%)	249 (98%)	147 (95%)	N.A.

^a^Solely based on the linguistic groups of Indo-Iranian, Niger-Congo, Semitic, and South Slavic languages because the HSCL-25 was not administered to participants in the linguistic group of Germanic languages; N.A. = Not available.

### 2.2. Measures

The HTQ (Mollica et al., 1992) is a self-report questionnaire assessing traumatic experiences and PTSD symptom severity. The HTQ consists of three parts of which only the second part is used in the present study. In the second part, severity of DSM-IV PTSD-symptoms is assessed by asking participants how much they were bothered by 16 PTSD-symptoms during the past week, rated on a 4-point scale (*not at all, a little bit, quite a bit*, or *extremely*). PTSD symptom severity is computed by averaging responses on the list of 16 PTSD-symptoms (range: 1–4). The HTQ recommends a clinical cut-off score of 2.5 to identify clinically significant PTSD.

The HSCL-25 (Mollica et al., 1996) is a self-report questionnaire assessing symptom severity with regard to anxiety and depression. Participants are asked to indicate how much they were bothered by 10 symptoms of anxiety and 15 symptoms of depression during the past week, rated on a 4-point scale (*not at all, a little bit, quite a bit*, or *extremely*). Symptom severity with regard to anxiety and depression is computed by averaging responses on the anxiety and depression items (range: 1–4). The HSCL-25 recommends a cut-off score of 1.75 to indicate clinically significant anxiety or depression.

The HTQ and the HSCL-25 are widely used with refugees and are available in many different languages (e.g. Amharic, Dari, English, French, Portuguese, Somali, Spanish, and Turkish). In addition, both instruments were translated into the most common languages spoken by refugees referred for treatment at Foundation Centrum ’45 (Arabic, Farsi, Serbo-Croatian, and Russian). Translations were carried out by certified translators or by bilingual staff members of Centrum ’45 and were reviewed by other certified translators (see Kleijn, Hovens, & Rodenburg, 2001). For the majority of individuals in each linguistic group, previously translated questionnaires could be used. Translations were, however, not available for all languages within the linguistic groups and interpreters were used for the minority of individuals in each linguistic group for whom no translated questionnaires were available.

### 2.3. Measurement invariance

In the present study MI is tested by a typical sequence of factor models with categorical factor indicators representing different levels of MI (for a detailed description see Millsap & Yun-Tein, 2004; Van Den Berg & Lance, 2000). The first level of MI, *configural invariance*, indicates that the construct under study is conceptualized in the same way by individuals from different groups (Steenkamp & Baumgartner, 1998). Configural invariance is met when the same factor structure holds across groups, but parameter estimates (i.e. factor loadings, thresholds, and residual variances) may vary across groups. When configural invariance is met, this does not mean that individuals respond in a similar way to the items, nor that cross-group comparisons of mean differences on the underlying construct are meaningful. This is captured by the second level of MI, *strong measurement invariance*, indicating that the strength of the relations between the items and the underlying construct is similar across groups, i.e. that individuals in different groups attribute the same meaning to the construct under study (Steenkamp & Baumgartner, 1998). It also implies that cross-group comparisons of mean differences on the underlying construct are meaningful. Strong MI holds when factor loadings and thresholds are equal across groups (Steenkamp & Baumgartner, 1998). The third and most stringent level of MI, *strict measurement invariance*, indicates that the underlying construct is measured identically across groups. If this level of MI is not met, cross-group comparisons of mean differences on the underlying construct are still meaningful, although means on the underlying construct are measured with different amount of error between groups (Steenkamp & Baumgartner, 1998; Van de Schoot, Lugtig, & Hox, 2012). Strict MI holds when factor loadings, thresholds, and residual variances are equal across groups (Steenkamp & Baumgartner, 1998). If a certain level of MI does not hold, the sequence of model testing stops.

Strong MI does not hold when one or more factor loadings or thresholds are not invariant across groups. It has been shown that when strong MI is not met, cross-group comparisons of latent (i.e. not observed) mean differences are still meaningful as long as strong MI holds for at least two items (Byrne, Shavelson, & Muthén, 1989). However, strong MI is necessary for cross-group comparisons of observed sum or mean scores on a scale (Van de Schoot, Lugtig, & Hox, 2012). If strong MI does not hold, it should be established whether there is partial MI. This can be done by scrutinizing parameter estimates and relaxing constraints on those factor loadings and thresholds that show substantive differences between groups (Steenkamp & Baumgartner, 1998; Van de Schoot, Lugtig, & Hox, 2012).

### 2.4. Statistical analyses

The software package MPlus Version 7.3 (Muthén & Muthén, 1998–2012) was used to establish the factor structure of the items of the HTQ and the HSCL-25 in the study sample in an exploratory factor analysis (EFA) for ordinal data with the weighted least squares means and variance adjusted (WLSMV) estimation. An underlying normal distribution was assumed for each item, where the four response categories were divided by three thresholds which were estimated from the data. Several models with different factor solutions were examined. Kaiser criterion (i.e. eigenvalues of the factors need to be larger than 1.0) and model fit statistics CFI, TLI, and RMSEA were used to assess the number of latent factors needed to adequately account for the correlation among item scores. The model with the best balance between model fit, parsimony, and interpretability was selected as the best factor model.

Subsequently, MPlus was used to conduct single and multigroup CFA to test different levels of MI of the HTQ and the HSCL-25 across linguistic groups. Configural invariance was tested by fitting the best factor models of the HTQ and the HSCL-25 from the EFA in a multigroup CFA of the total sample and in single-group CFAs for each of the linguistic groups separately. In the multigroup CFA, factor loadings and thresholds were freely estimated across linguistic groups, and residual variances were fixed at one in all groups. Strong MI was tested by fitting a multigroup CFAs in which factor loadings and thresholds were constrained to be equal. Residual variances were fixed at one in the first group and freely estimated in the other groups. It was tested whether the fit of the model representing strong MI was better compared to the model representing configural invariance. Partial strong MI was tested by relaxing equality constraints on factor loadings and thresholds for those items that showed substantive cross-group differences with regard to factor loadings and/or thresholds. It was tested whether the fit of the model representing partial strong MI was better compared to the model representing configural invariance. Strict MI was assessed by fixing residual variances at one across those groups in which factor loadings and thresholds were allowed to be constrained to be equal. It was tested whether this model fit the data better compared to the model representing (partial) strong MI.

Single and multigroup CFAs with categorical factor indicators were estimated with the WLSMV estimator using the THETA parameterization. CFI, TLI, and RMSEA were used to assess model fit. For CFI and TLI, model fit is considered good when values are close to .95 (Hu & Bentler, 1999). It must be noted that TLI is sensitive to small sample sizes (Van de Schoot, Lugtig, & Hox, 2012). RMSEA is considered adequate when the value is < .08 and good when it is < .05 (Browne & Cudeck, 1993; Schermelleh-Engel & Moosbrugger, 2003). The difference in goodness-of-fit between nested MI models was evaluated by the χ^2^ difference test and the difference in CFI between two nested models. The ‘difftest’ option in MPlus was used for appropriate χ^2^ difference testing with the WLSMV estimator (Muthén & Muthén, 1998–2012). The χ^2^ difference test is highly sensitive to sample size such that even trivial differences between two nested models may be significant (Cheung & Rensvold, 2002). As an alternative, it has been suggested to interpret the χ^2^ difference test by the ratio of the χ^2^ value and the number of estimated parameters (χ^2^/df). A χ^2^/df ratio of less than 3 indicates a better fit of the nested model compared to the more complex model (Schermelleh-Engel & Moosbrugger, 2003). A difference in CFI < 0.01 also indicates a better fit of the nested model compared to the more complex model (Cheung & Rensvold, 2002).

## 3. Results

Symptom severity with regard to PTSD, anxiety, and depression for the total sample, as well as the five linguistic groups, is presented in the lower part of Table 1. Mean severity level of posttraumatic stress symptoms was 2.9 in the total sample, with 74% of participants being symptomatic for posttraumatic stress disorder (ranging between 49% in the linguistic group of Germanic languages and 89% in the linguistic group of Semitic languages). Mean symptom severity with regard to anxiety and depression was 2.9 in the total sample, with 94% and 96% being symptomatic for anxiety and depressive disorder respectively. Differences in the number of participants being symptomatic for anxiety and depressive disorder between the linguistic groups were small.

### 3.1. EFA and MI analysis of the HTQ

First, EFA was conducted on the total sample in order to establish the factor structure of the HTQ in the present sample. Based on model fit and eigenvalues, EFA yielded a 3-factor solution as a good fit for the 16 items of the HTQ. Table 2 presents the unstandardized Geomin rotated factor loadings and eigenvalues of the 3-factor solution. CFI (.980) and TLI (.968) indicated good model fit and RMSEA (.060) indicated adequate model fit. Eigenvalues of the three factors were larger than one whereas eigenvalues of the fourth to sixteenth factor were lower than one (i.e. ranging between .199 and .785). The items that cluster on the same factor suggest that the first factor (items 1, 2, 3, 8, and 16) reflects symptoms of intrusion, the second factor (items 4, 5, 6, 7, 9, 10, 13, and 14) hypervigilance, and the third factor (items 11, 12, and 15) avoidance.

**Table 2.** Geomin rotated factor loadings and eigenvalues of the 3-factor model of the HTQ as estimated by EFA.

	F1	F2	F3
1. Recurrent thoughts or memories of the most hurtful or terrifying events	.**98**	−.15	.01
2. Feeling as though the event is happening again	.**85**	−.02	−.01
3. Recurrent nightmares	.**79**	.01	.04
4. Feeling detached or withdrawn from people	−.02	.**70**	.01
5. Unable to feel emotions	.00	.**54**	.07
6. Feeling jumpy, easily startled	.**32**	.**52**	−.01
7. Difficulty concentrating	.26	.**51**	−.02
8. Trouble sleeping	.**63**	.19	.00
9. Feeling on guard	.18	.**46**	.06
10. Feeling irritable or having outburst of anger	.25	.**46**	−.01
11. Avoiding activities that remind you of the hurtful or terrifying events	.26	.00	.**57**
12. Inability to remember parts of the hurtful or terrifying events	−.19	.19	.**36**
13. Less interest in daily activities	.12	.**65**	.01
14. Feeling as if you don’t have a future	.25	.**54**	.03
15. Avoiding thoughts or feelings associated with the hurtful of terrifying events	.01	−.15	**1.02**
16. Sudden emotional or physical reaction when reminded of the traumatic events	.**54**	.03	.**31**
Eigenvalues	7.762	1.300	1.039

Factor loadings over .30 appear in bold; Model fit: χ^2^ = 415.797, df = 75, CFI = .980, TLI = .968, RMSEA = .060.

Table 3 presents model fitting results of the MI analysis across five linguistic groups that was conducted on the 3-factor model for PTSD that resulted from the EFA on the items of the HTQ. In model 1, a multigroup 3-factor model of PTSD was tested. Factor loadings and thresholds were freely estimated in each of the linguistic groups. CFI and TLI indicated good model fit, and RMSEA indicated adequate model fit. Model 1a–1e tested the 3-factor model of PTSD for each of the linguistic groups separately. CFI and RMSEA indicated that the model fit in each of the linguistic groups was adequate to good. TLI indicated that the model fit was good for the Indo-Iranian, Niger-Congo, and Germanic language group but not for the Semitic and South-Slavic language group since it deviated substantially from .95. It must be noted that TLI is sensitive to small samples and the actual sample sizes of the individual linguistic groups are relatively small. Because model fit indices of the multigroup 3-factor model for PTSD representing configural MI (model 1) were adequate to good and model fit indices of the 3-factor model for PTSD in each of the relatively small linguistic groups were mainly adequate it was concluded that configural invariance holds for the HTQ across five linguistic groups.

**Table 3.** Model fitting results for testing MI of the Harvard Trauma Questionnaire across five linguistic groups.

	vs.	χ^2^	df	∆χ^2^	∆df	χ^2^/df	CFI	∆CFI	TLI	RMSEA
1. Configural MI: total sample	–	1072.991	505	–	–	–	.962	–	.955	.067
1a. Configural MI: Indo-Iranian languages	–	220.150	101	–	–	–	.950	–	.941	.067
1b. Configural MI: Niger-Congo languages	–	119.752	101	–	–	–	.985	–	.982	.038
1c. Configural MI: Semitic languages	–	219.875	101	–	–	–	.922	–	.908	.064
1d. Configural MI: South Slavic languages	–	243.923	101	–	–	–	.924	–	.908	.085
1e. Configural MI: Germanic languages	–	290.933	101	–	–	–	.976	–	.972	.071
2. Strong MI	1	1429.417	673	414.764	168	2.469	.949	.013	.955	.067
**3. Partial strong MI**	**1**	**1189.434**	**631**	**188.134**	**126**	**1.493**	.**963**	**−.001**	.**964**	.**060**
4. Partial strict MI	3	1303.349	679	146.734	48	3.057	.958	.005	.963	.061

Best fitting model is printed in bold; vs. = versus; χ^2^, df = chi-square test statistic and degrees of freedom for model; ∆χ^2^, ∆df = chi-square test statistic and degrees of freedom for chi-square difference test between two nested models; χ^2^/df = ratio between χ^2^ and degrees of freedom with regard to the chi-square difference test; ∆CFI = difference in CFI value between two nested models.

Model 2 tested the multigroup 3-factor model of PTSD representing strong MI. Factor loadings and thresholds were constrained to be equal across five linguistic groups. CFI and TLI indicated good model fit and RMSEA indicated adequate model fit. The χ^2^/df ratio indicated that the fit of model 2 was not worse compared to model 1. The difference in CFI between model 1 and model 2 indicated a worse fit of model 2 compared to model 1.

Factor loadings and thresholds (see Supplementary Tables S1 and S2) were scrutinized to investigate possible differences between linguistic groups. Thresholds appeared to differ substantively between the Germanic language group and the other linguistic groups, indicating that participants in the Germanic language group systematically reported milder symptom severity on the items of the HTQ. No systematic differences with regard to factor loadings appeared. Model 3 tested a multigroup 3-factor model of PTSD representing partial strong MI. Factor loadings and thresholds were constrained to be equal in the Indo-Iranian, Niger-Congo, Semitic, and South Slavic language groups and were freely estimated in the Germanic language group. CFI and TLI indicated good model fit and RMSEA indicated adequate model fit. The χ^2^/df ratio, as well as the difference in CFI between model 1 and model 3, indicated that the fit of model 3 was not worse than model 1. Therefore, model 3 is preferred over model 1 and model 2 and it can be concluded that partial strong MI invariance holds for the HTQ across linguistic groups. More specifically, strong MI held across the Indo-Iranian, Niger-Congo, Semitic, and South Slavic language groups, but not for the Germanic language group.

Model 4 tested a multigroup 3-factor model of PTSD representing partial strict MI. Factor loadings and thresholds were constrained to be equal, and residual variances were fixed at one in the Indo-Iranian, Niger-Congo, Semitic, and South Slavic language group. In the Germanic language group, factor loadings and thresholds were freely estimated and residual variances were fixed at one. CFI and TLI indicated good model fit and RMSEA indicated adequate fit. The χ^2^/df ratio was slightly larger than the cut-off value of 3, indicating that the fit of model 4 was worse compared to model 3. The difference in CFI between model 3 and model 4 indicated that the fit of model 4 was not worse compared to model 3. Based on the goodness-of-fit indexes model 3 was preferred over model 4. It was therefore concluded that partial strict MI does not hold across the Indo-Iranian, Niger-Congo, Semitic, and South Slavic language groups, and that model 3 representing partial strong MI fit the data best.

### 3.2. EFA and MI analysis of the HSCL-25

EFA on the total sample was first conducted to establish the factor structure of the HSCL-25 in the present sample. Based on model fit and eigenvalues, EFA yielded a 3-factor solution as a good fit for the 25 items of the HSCL-25. CFI (.973), TLI (.964), and RMSEA (.044) indicated good model fit. Eigenvalues of the three factors were larger than one, whereas eigenvalues of the fourth to twenty-fifth factor were lower than one (i.e. ranging between .202 and .968). Table 4 presents the unstandardized Geomin rotated factor loadings and eigenvalues of the 2-factor and 3-factor solution. With regard to the 3-factor solution, it can be seen that items clustering on the first and second factor highly overlap, indicating that the first and second factor are insufficiently distinctive. Therefore, the 2-factor solution was preferred over the 3-factor solution. CFI (.942), TLI (.931), and RMSEA (.062) indicated adequate model fit for the 2-factor solution, and eigenvalues were larger than one. A low factor loading (λ = .28) was observed for item 8 (‘Headaches’) on the first and second factor, indicating that this item does not add substantively to both factors. EFA was therefore rerun without item 8 and yielded a 2-factor solution with adequate fit (CFI = .944, TLI = .933, RMSEA = .062) and eigenvalues larger than one for the 24 remaining items of the HSCL-25. This model was selected as the best model. The items that cluster on the same factor suggest that the first factor (items 1–7 and 9–10) reflects symptoms of anxiety and the second factor (items 11–25) represents symptoms of depression.

**Table 4.** Geomin rotated factor loadings and eigenvalues of the 3- and 2-factor model of the HSCL-25 as estimated by EFA.

	3-factor solution			2-factor solution	
	F1	F2	F3	F1	F2
1. Suddenly scared for no reason	.**85**	.01	.01	.**89**	−.12
2. Feeling fearful	.**78**	−.02	.09	.**81**	−.04
3. Faintness, dizziness or weakness	.16	.**60**	.08	.**41**	.28
4. Nervousness or shakiness inside	.**47**	.**47**	.01	.**67**	.13
5. Heart pounding or racing	.**43**	.**43**	−.03	.**61**	.08
6. Trembling	.**45**	.**51**	−.13	.**67**	.01
7. Feeling tense or keyed up	.**32**	.**44**	.11	.**50**	.24
8. Headaches	.11	.**38**	.16	–	–
9. Spells of terror or panic	.**65**	.12	.11	.**70**	.07
10. Feeling restless, can’t sit still	.**40**	.18	.19	.**47**	.21
11. Feeling low in energy, slowed down	−.01	.**60**	.23	.27	.**43**
12. Blaming yourself for things	.01	.02	.**53**	−.01	.**56**
13. Crying easily	.12	.17	.27	.18	.**32**
14. Loss of sexual interest or pleasure	−.02	.28	.**37**	.09	.**47**
15. Poor appetite	−.08	.25	.**35**	.02	.**45**
16. Difficulty falling asleep, staying asleep	.10	.28	.**39**	.20	.**48**
17. Feeling hopeless about the future	.12	−.13	.**79**	.01	.**76**
18. Feeling blue	.10	.00	.**76**	.06	.**76**
19. Feeling lonely	−.05	.01	.**69**	−.09	.**72**
20. Thoughts of ending your life	.18	−.05	.**58**	.11	.**56**
21. Feeling of being trapped or caught	.08	.07	.**65**	.06	.**68**
22. Worrying too much about things	.05	.15	.**55**	.08	.**61**
23. Feeling no interest in things	−.11	.10	.**72**	−.11	.**79**
24. Feeling everything is an effort	−.10	.26	.**64**	−.01	.**75**
25. Feelings of worthlessness	.02	−.05	.**75**	−.06	.**76**
Eigenvalues	10.06	1.93	1.31	9.82	1.93

Factor loadings over .30 appear in bold; Model fit 3-factor solution: χ^2^ = 569,968, df = 228, CFI = .973, TLI = .964, RMSEA = .044; Model fit 2-factor solution: χ^2^ = 900.549, df = 229, CFI = .944, TLI = .933, RMSEA = .062.

Table 5 presents model fitting results of the MI analysis across four linguistic groups that was conducted on the 2-factor model of anxiety and depression that resulted from the EFA on the items of the HSCL-25. In model 1, a multigroup 2-factor model of anxiety and depression was tested. Factor loadings and thresholds were freely estimated in each of the linguistic groups. CFI, TLI, and RMSEA indicated adequate model fit. In model 1a–1d, the 2-factor model of anxiety and depression was tested for each of the linguistic groups separately. All fit indices indicated adequate model fit in each of the subsamples. Based on the model fitting results of model 1 and model 1a–1d it can be concluded that configural invariance holds for the HSCL-25 across four linguistic groups.

**Table 5.** Model fitting results for testing MI of the Hopkins Symptom Checklist-25 across four linguistic groups.

	vs.	χ^2^	df	∆χ^2^	∆df	χ^2^/df	CFI	∆CFI	TLI	RMSEA
1. Configural MI: total sample	–	1672.508	1004	–	–	–	.945	–	.939	.059
1a. Configural MI: Indo-Iranian languages	–	495.364	251	–	–	–	.939	–	.933	.067
1b. Configural MI: Niger-Congo languages	–	345.402	251	–	–	–	.951	–	.946	.055
1c. Configural MI: Semitic languages	–	467.418	251	–	–	–	.939	–	.933	.058
1d. Configural MI: South Slavic languages	–	383.224	251	–	–	–	.952	–	.947	.058
2. Strong MI	1	2007.550	1208	415.521	204	2.037	.934	.011	.940	.059
**3. Partial strong MI**	**1**	**1990.385**	**1199**	**395.194**	**195**	**2.027**	.**935**	.**010**	.**940**	.**059**

Best fitting model is printed in bold; vs. = versus; χ^2^, df = chi-square test statistic and degrees of freedom for model; ∆χ^2^, ∆df = chi-square test statistic and degrees of freedom for chi-square difference test between two nested models; χ^2^/df = ratio between χ^2^ and degrees of freedom with regard to the chi-square difference test; ∆CFI = difference in CFI value between two nested models.

Model 2 tested the multigroup 2-factor model of anxiety and depression representing strong MI. Factor loadings and thresholds were constrained to be equal across groups. CFI, TLI, and RMSEA indicated adequate model fit. The χ^2^/df ratio indicated that the fit of model 2 was not worse compared to model 1. The difference in CFI between model 1 and model 2 indicated a worse fit of model 2 compared to model 1.

Factor loadings and thresholds (see Supplementary Tables S3 and S4) were subsequently scrutinized to investigate possible differences between linguistic groups. No systematic differences with regard to factor loadings and thresholds were observed. Differences between linguistic groups were generally small, with the exception of factor loadings and thresholds regarding item 4 (Nervousness or shakiness inside). Model 3 tested a multigroup 2-factor model of anxiety and depression representing partial strong MI. In this model, factor loadings and thresholds with regard to item 4 were freely estimated across linguistic group whereas all other factor loadings and thresholds were constrained to be equal. CFI, TLI, and RMSEA indicated adequate model fit. The χ^2^/df ratio, as well as the difference in CFI between model 1 and model 3, indicated that the fit of model 3 was not worse than model 1. Therefore, model 3 is preferred over model 1 and model 2 and it can be concluded that partial strong MI invariance holds for the HSCL-25 across linguistic groups.

## 4. Discussion

### 4.1. Overview

This study investigated the factor structure and MI of two widely used instruments for the assessment of PTSD symptoms (HTQ) and symptoms of anxiety and depression (HSCL-25) among Dutch and refugee patients with different linguistic backgrounds. EFA yielded a 3- and a 2-factor structure for the items of the HTQ and the HSCL-25, respectively. In addition, MI analyses on the HTQ showed strong MI across the groups of refugee patients with Indo-Iranian, Niger-Congo, Semitic, and South Slavic language backgrounds. Strong MI could, however, not be demonstrated across the group of Dutch patients with a Germanic language background and the groups of refugee patients with non-western language backgrounds. MI analyses on the HSCL-25 indicated partial strong MI across the four non-western linguistic groups of refugee patients.

### 4.2. Factor structure and MI of the HTQ

Armour and colleagues (2016) stated that consensus regarding the exact number and nature of factors is yet to be reached, despite numerous studies on the factor structure of PTSD. We found a 3-factor solution in which the items of the HTQ of symptoms of intrusions were represented by the first factor, symptoms of hypervigilance by the second factor, and symptoms of avoidance by the third factor in line with the DSM-IV criteria of PTSD. Armour and colleagues (2016) showed that 4-factor models received substantial support (e.g. Elklit & Shevlin, 2007; Palmieri, Marshall, & Schell, 2007), but the 5-factor dysphoric arousal model demonstrated the best fit (e.g. Charak et al., 2014). Thus, in contrast to our results, most studies provided evidence for the recently proposed DSM-5 PTSD model (e.g. Fodor et al., 2015; Vindbjerg, Carlsson, Mortensen, Elklit, & Makransky, 2016).

In MI analyses, we showed that posttraumatic stress as measured by the HTQ is conceptualized by symptoms of intrusion, hypervigilance, and avoidance by Dutch patients with a Germanic language background as well as by refugee patients with Indo-Iranian, Niger-Congo, Semitic, and South Slavic language backgrounds (i.e. configural invariance). Dutch patients reported milder symptom severity on most items of the HTQ. This result is consistent with previous findings that immigrants tend to report higher levels of complaints on questionnaires than the dominant group in the host country (He & Van De Vijver, 2013; Morren, Gelissen, & Vermunt, 2012). Differences of observed scale scores between Dutch patients and refugee patients with non-western language backgrounds either reflect measurement bias instead of true underlying differences in PTSD symptom severity (Meredith, 1993; Van de Schoot, Lugtig, & Hox, 2012) or reflect the notion that refugees score higher on PTSD as a result of experiencing more traumatic events (e.g. de Jong et al., 2001). We conclude that it is advisable to develop differentiated cut-off scores with regard to the HTQ for patients with a western language background and for refugee patient groups with non-western language backgrounds.

In contrast, strong MI of the HTQ was demonstrated across the groups of refugee patients with Indo-Iranian, Niger-Congo, Semitic, and South Slavic language backgrounds. This means that the items of the HTQ as well as the concepts they are measuring (i.e. the PTSD symptom dimensions of intrusion, hypervigilance, and avoidance) are interpreted in the same way by refugee patients with different non-western linguistic backgrounds (Horn & McArdle, 1992; Van de Schoot, Lugtig, & Hox, 2012). Therefore, meaningful comparisons of observed PTSD scale scores on the HTQ between refugee patients with different non-western linguistic backgrounds can be made. Likewise, the use of a single PTSD cut-off score with regard to the HTQ in groups of refugee patients with different non-western linguistic backgrounds seems feasible.

### 4.3. Factor structure and MI of the HSCL-25

According to Al-Turkait and colleagues (2011), most scholars found evidence for the 2-factor model and the 3-factor model of the HSCL-25. The 2-factor model comprises symptoms specific to anxiety and symptoms specific to depression, and the 3-factor model additionally distinguishes nonspecific symptoms of general distress which the two disorders share (Al-Turkait et al., 2011). Glaesmer and colleagues (2013) concluded that, because of the high inter-correlations of the factors of the tripartite model, the 2-factor model is the preferable factor solution. Similarly, we found that the HSCL-25 was represented best by a 2-factor model comprising symptoms of anxiety and symptoms of depression. Although research showed that headaches are usually part of the anxiety scale (e.g. Al-Turkait et al., 2011; Glaesmer et al., 2013) or at least coincide with depression and anxiety (Juang, Wang, Fuh, Lu, & Su, 2000; Zwart et al., 2003), in our non-western refugee groups headache was part of neither the depression nor the anxiety scale. This indicates that among non-western refugee groups headache is not part of depression or anxiety.

In addition, MI analyses indicated that it can be concluded that anxiety and depression items and the underlying constructs as measured with the HSCL-25 are interpreted in the same way by refugee patients with different non-western linguistic backgrounds, with the exception of one item (i.e. Nervousness or shakiness inside) regarding the anxiety construct to which they appeared to respond differently. Cross-group comparisons of observed anxiety scores are only meaningful when the non-invariant item is discarded. Yet, the question remains whether the commonly used cut-off score for anxiety with regard to the HSCL-25 applies to this scale.

Previous studies have focused on configural invariance to examine whether screening outcomes can be compared across linguistic or cultural groups (e.g. Fodor et al., 2015), providing evidence for conceptual similarity of mental health concepts are. We conclude, in line with these previous findings, that depression, anxiety, and posttraumatic stress are conceptually similar across our groups under study. The strength of our study is that it is one of the very few that examined strong and strict MI as well beyond this traditional and common question of configural invariance (see also Rasmussen et al., 2015). Based on our findings we conclude that mental health questionnaires do indeed help clinicians in their fundamental task to target symptoms and assess treatment outcomes among refugees. Since our results suggested that PTSD, anxiety, and depression are conceptualized in a similar way by groups of refugees with different non-western linguistic backgrounds, and that they interpret items with regard to these concepts in the same way, it can be concluded that local idioms of distress and inherent cultural response patterns may not play a major role when using the HTQ and the HSCL-25. Future studies need to examine whether the commonly used cut-off scores with regard to both questionnaires apply for refugee patients with non-western linguistic backgrounds. We add that ideally one must carefully make an inventory of the expression of distress in other languages before one can conclude that the way people perceive their problem may or may not overlap DSM-IV or DSM-5 categories. Local categories of emotional distress help place the instruments within their proper cultural context (Bolton & Tang, 2002; de Jong, 2002).

### 4.4. Limitations

One limitation is that we did not conduct the HSCL-25 to Dutch patients. Consequently, we could not compare the non-western groups of refugees with a western group, whereas our findings on the HTQ found different thresholds for the Dutch groups compared to the non-western groups of refugees. Another limitation is that the linguistic groups differ in sample size and this may have biased the outcomes of our multigroup CFA. A simulation study of Meade and Bauer (2007) indicated that the precision of estimated factor loading differences is high for sample sizes of 400, but varied somewhat by condition at sample sizes of 100 and 200. Since sample sizes of all the linguistic groups were smaller than 400, this may have biased our outcomes.

### 4.5. Conclusion

Because of the huge number of refugees that currently cross the European borders (UNHCR, 2015), of whom most are severely traumatized, there is a need to detect those who suffer from psychological complaints to be able to meet their mental health needs. Our study results indicate that the HTQ and the HSCL-25 can be useful in this respect. They can be applied in non-western refugee patient populations. Local idioms of distress and response patterns may not play a major role when using the HTQ and the HSCL-25 among non-western refugee patients.

Future studies need to examine whether the used cut-off scores with regard to both questionnaires need to be reconsidered for refugee patients with non-western linguistic backgrounds. Although meaningful comparisons of observed PTSD and depression scores between groups of refugee patients with different non-western linguistic background are feasible, comparisons between patients with a western and non-western linguistic background, as well as comparisons of anxiety scores, are likely to be biased.

This study is one of the few to test different levels of MI and provide evidence for partial MI of mental health questionnaires among non-western refugees, yielding a discerned answer to the construct validity question of mental health concepts among refugees. As such, this research is an invitation – and perhaps a roadmap – for future researchers to further test these findings.

## Supplementary Material

Supplementary material

## Highlights

- We conclude that mental health constructs of PTSD, anxiety, and depression, as measured by the HTQ and the HSCL-25, are to a large extent interpreted in a similar way by refugee patients.
- Local idioms of distress and inherent response patterns may not play a major role when applying the HTQ and the HSCL-25 in non-western refugee patient populations.
- Our study is one of the few to provide evidence for (partial) strong measurement invariance of mental health screeners among refugees.

## References

[CIT0001] Al-Turkait F. A., Ohaeri J. U., El-Abbasi A.-H. M., & Naguy A. (2011). Relationship between symptoms of anxiety and depression in a sample of Arab college students using the Hopkins Symptom Checklist-25. *Psychopathology*, 44, 230–241. doi:10.1159/000322797 21502775

[CIT0002] Armour C., Műllerová J., & Elhai J. D. (2016). A systematic literature review of PTSD’s latent structure in the diagnostic and statistical manual of mental disorders: DSM-IV to DSM-5. *Clinical Psychology Review*, 44, 60–74. doi:10.1016/j.cpr.2015.12.003 26761151

[CIT0003] Bolton P., & Tang A. M. (2002). An alternative approach to cross-cultural function assessment. *Social Psychiatry and Psychiatric Epidemiology*, 37(11), 537–543. doi:10.1007/s00127-002-0580-5 12395144

[CIT0004] Browne M. W., & Cudeck R. (1993). Alternative ways of assessing model fit. *Sociological Methods and Research*, 21, 230–239. doi:10.1177/0049124192021002005

[CIT0005] Buhman C, Mortensen E.L, Lundstrøm S, Ryberg J, Nordentoft M, & Ekstrøm M. (2014). Symptoms. *Quality of Life and Level of functioning of traumatized refugees at psychiatric trauma clinic in Copenhagen. Torture*, 24(1), 25-39. 25590462

[CIT0006] Byrne B. M., Shavelson R. J., & Muthén B. O. (1989). Testing for the equivalence of factor covariance and mean structures: The issue of partial measurement invariance. *Psychological Bulletin*, 105, 456–466. doi:10.1037/0033-2909.105.3.456

[CIT0007] Charak R., Armour C., Elklit A., Angmo D., Elhai J. D., & Koot H. M. (2014). Factor structure of PTSD, and relation with gender in trauma survivors from India. *European Journal of Psychotraumatology*, 5(1), 25547. doi:10.3402/ejpt.v5.25547 25413575 PMC4247496

[CIT0008] Cheung G. W., & Rensvold R. B. (2002). Evaluating goodness-of-fit indexes for testing measurement invariance. *Structural Equation Modeling: A Multidisciplinary Journal*, 9(2), 233–255. doi:10.1207/S15328007SEM0902_5

[CIT0009] Contractor A. A., Claycomb M. A., Byllesby B. M., Layne C. M., Kaplow J. B., Steinberg A. M., & Elhai J. D. (2015). Hispanic ethnicity and Caucasian race: Relations with posttraumatic stress disorder’s factor structure in clinic-referred youth. *Psychological Trauma : Theory, Research, Practice and Policy*, 7(5), 456–464. doi:10.1037/tra0000068 26147448

[CIT0010] de Jong J. T., Komproe I. H., & Van Ommeren M. (2003). Common mental disorders in postconflict settings. *Lancet*, 361, 2128–2130. doi:10.1016/S0140-6736(03)13692-6 12826440

[CIT0011] de Jong J. T., Komproe I. H., Van Ommeren M., El Masri M., Araya M., Khaled N., … Somasundaram D. (2001). Lifetime events and posttraumatic stress disorders in 4 postconflict settings. *Jama*, 286(5), 555–562. doi:10.1001/jama.286.5.555 11476657

[CIT0012] de Jong J. T. V. M. (2002). *Trauma, war, and violence: Public mental health in socio-cultural context*. New York: Plenum.

[CIT0013] Dyer N. G., Hanges P. J., & Hall R. J. (2005). Applying multilevel confirmatory factor analysis techniques to the study of leadership. *The Leadership Quarterly*, 16, 149–167. doi:10.1016/j.leaqua.2004.09.009

[CIT0014] Elklit A., & Shevlin M. (2007). The structure of PTSD symptoms: A test of alternative models using confirmatory factor analysis. *The British Journal of Clinical Psychology*, 46(Pt 3), 299–313. doi:10.1348/014466506X171540 17535523

[CIT0015] Fodor K. E., Pozen J., Ntaganira J., Sezibera V., & Neugebauer R. (2015). The factor structure of posttraumatic stress among Rwandans exposed to the 1994 genocide: A confirmatory factor analytic study using PCL-C. *Journal of Anxiety Disorders*, 32, 8–16. doi:10.1016/j.janxdis.2015.03.001 25840139

[CIT0016] Glaesmer H., Braehler E., Grande G., Hinz A., Petermann F., & Romppel M. (2013). The German version of the Hopkins Symptom Checklist (HSCL-25) – factorial structure, psychometric properties, and population-based norms. *Comprehensive Psychiatry*, 55(2), 396–403. doi:10.1016/j/comppsych.2013.08.020 24286991

[CIT0017] Hall B., & Olff M. (2016). Global mental health: Trauma and adversity among populations in transition. *European Journal Of Psychotraumatology*, 7, 31140. doi:10.3402/ejpt.v7.31140 26886490 PMC4756626

[CIT0018] Hassan G., Ventevogel P., Jefee-Bahloul H., Barkil-Oteo A., & Kirmayer L. J. (2016). Mental health and psychosocial wellbeing of Syrians affected by armed conflict. *Epidemiology and Psychiatric Sciences*, 1, 1–13. doi:10.1017/S2045796016000044 PMC6998596 26829998

[CIT0019] He J., & Van De Vijver F. J. R. (2013). A general response style factor: Evidence from a multi-ethnic study in the Netherlands. *Personality and Individual Differences*, 55, 794–800. doi:10.1016/j.paid.2013.06.017

[CIT0020] Hollifield M., Verbillis-Kolp S., Farmer B., Toolson E. C., Woldehaimanot T., Yamazaki J., … SooHoo J. (2013). The refugee health screener-15 (RHS-15): Development and validation of an instrument for anxiety, depression, and PTSD in refugees. *General Hospital Psychiatry*, 35(2), 202–209. doi:10.1016/j.genhosppsych.2012.12.002 23347455

[CIT0021] Horn J., & McArdle J. J. (1992). A practical and theoretical guide to measurement invariance in aging research. *Experimental Aging Research*, 18, 117–144. doi:10.1080/03610739208253916 1459160

[CIT0022] Hu L., & Bentler P. M. (1999). Cutoff criteria for fit indexes in covariance structure analysis: Conventional criteria versus new alternatives. *Structural Equation Modeling: A Multidisciplinary Journal*, 6(1), 1–55. doi:10.1080/10705519909540118

[CIT0023] Juang K.-D., Wang S.-J., Fuh J.-L., Lu S.-R., & Su T.-P. (2000). Comorbidity of depressive and anxiety disorders in chronic daily headache and its subtypes. *Headache: The Journal of Head and Face Pain*, 40(10), 818–823. doi:10.1046/j.1526-4610.2000.00148.x 11135026

[CIT0024] Kaiser B. N., Haroz E. E., Kohrt B. A., Bolton P. A., Bass J. K., & Hinton D. E. (2015). “Thinking too much”: A systematic review of a common idiom of distress. *Social Science & Medicine (1982)*, 147, 170–183. doi:10.1016/j.socscimed.2015.10.044 26584235 PMC4689615

[CIT0025] Katzner K. (2002). *Languages of the world* (3rd ed.). London: Routledge.

[CIT0026] Kleijn W. C., Hovens J. E., & Rodenburg J. J. (2001). Posttraumatic stress symptoms in refugees: Assessments with the Harvard Trauma Questionnaire and the Hopkins Symptom Checklist-25 in different languages. *Psychological Reports*, 88, 527–532. doi:10.2466/pr0.2001.88.2.527 11351903

[CIT0027] Meade A. W., & Bauer D. J. (2007). Power and precision in confirmatory factor analytic tests of measurement invariance. *Structural Equation Modeling: A Multidisciplinary Journal*, 14(4), 611–635. doi:10.1080/10705510701575461

[CIT0028] Meredith W. (1993). Measurement invariance, factor analysis, and factorial invariance. *Psychometrika*, 58, 525–543. doi:10.1007/BF02294825

[CIT0029] Miller K. E., Kulkarni M., & Kushner H. (2007). Beyond trauma-focused psychiatric epidemiology: Bridging research and practice with war-affected populations. *American Journal of Orthopsychiatry*, 76(4), 409–422. doi:10.1037/0002-9432.76.4.409 17209709

[CIT0030] Millsap R. E., & Yun-Tein J. (2004). Assessing factorial invariance in ordered-categorical measures. *Multivariate Behavioral Research*, 39(3), 479–515. doi:10.1207/S15327906MBR3903_4

[CIT0031] Mollica R. F., Caspi-Yavin Y., Bollini P., Truong T., Tor S., & Lavelle J. (1992). The Harvard Trauma Questionnaire. Validating a cross-cultural instrument for measuring torture, trauma, and posttraumatic stress disorder in Indochinese refugees. *The Journal of Nervous and Mental Disease*, 180, 111–116. 1737972

[CIT0032] Mollica R.F., Caspi-Yavin Y., Lavelle J., Tor S., Yang T., Chan S., ... de Marneffe D. (1996) Symptom Checklist (HSCL-25): Manual for Cambodian, Laotian and Vietnamese versions. Torture, 6, 35–42. doi: 10.12807/ti.108202.2016.a07

[CIT0033] Morren M., Gelissen J. P. T. M., & Vermunt J. K. (2012). Response strategies and response styles in cross-cultural surveys. *Cross-Cultural Research*, 46, 255–279. doi:10.1177/1069397112440939

[CIT0034] Muthén L. K., & Muthén B. O. (1998–2012). *MPlus user’s guide* (7th ed.). Los Angeles, CA: Muthén & Muthén.

[CIT0035] Narrow W. E., Rae D. S., Robins L. N., & Regier D. A. (2002). Revised prevalence estimates of mental disorders in the United States: Using a clinical significance criterion to reconcile 2 surveys’ estimates. *Arch.Gen.Psychiatry*, 59(2), 115–123. 11825131 10.1001/archpsyc.59.2.115

[CIT0036] Olff M. (2015). Choosing the right instruments for psychotrauma related research. *European Journal of Psychotraumatology*, 6, 30585. doi:10.3402/ejpt.v6.30585 PMC4696459 26714933

[CIT0037] Palmieri P. A., Marshall G. N., & Schell T. L. (2007). Confirmatory factor analysis of posttraumatic stress symptoms in Cambodian refugees. *Journal of Traumatic Stress*, 20, 207–216. doi:10.1002/jts.20196 17427910

[CIT0038] Poortinga Y. H. (1975). Limitations on intercultural comparison of psychological data. *Nederlands Tijdschrift Voor De Psychologie En Haar Grensgebieden*, 30, 23–39.

[CIT0039] Purgato M., & Olff M. (2015). Global mental health and trauma: The current evidence and the long road ahead. *European Journal Of Psychotraumatology*, 6, 30120. doi:10.3402/ejpt.v6.30120 26589260 PMC4654766

[CIT0040] Rasmussen A., Verkuilen J., Ho E., & Fan Y. (2015). Posttraumatic stress disorder among refugees: Measurement invariance of Harvard Trauma Questionnaire Scores across global regions and response patterns. *Psychological Assessment*, 27(4), 1160–1170. doi:10.1037/pas0000115 25894706 PMC4615261

[CIT0041] Schermelleh-Engel K., & Moosbrugger H. (2003). Evaluating the fit of structural equation models: Tests of significance and descriptive goodness-of-fit measures. *Methods of Psychological Research Online*, 8(2), 23–74.

[CIT0042] Schnyder U., Müller J., Morina N., Schick M., Bryant R. A., & Nickerson A. (2015). A comparison of DSM-5 and DSM-IV diagnostic criteria for posttraumatic stress disorder in traumatized refugees. *Journal of Traumatic Stress*, 28(4), 267–274. doi:10.1002/jts.22023 26194738

[CIT0043] Steenkamp J.-B. E. M., & Baumgartner H. (1998). Assessing measurement invariance in cross-national consumer research. *Journal of Consumer Research*, 25, 78–90. doi:10.1086/209528

[CIT0044] Sweetland A. C., Belkin G. S., & Verdeli H. (2014). Measuring depression and anxiety in sub-Saharan Africa. *Depression and Anxiety*, 31(3), 223–232. doi:10.1002/da.22142 23780834 PMC4109689

[CIT0045] UNHCR (2015). 2015 regional operations profile - Europe. Retrieved from http://www.unhcr.org/pages/4a02d9346.html

[CIT0046] Van de Schoot R., Lugtig P., & Hox J. (2012). A checklist for testing measurement invariance. *European Journal of Developmental Psychology*, 9(4), 486–492. doi:10.1080/17405629.2012.686740

[CIT0047] Van Den Berg R. J., & Lance C. E. (2000). A review and synthesis of the measurement invariance literature: Suggestions, practices, and recommendations for organizational research. *Organizational Research Methods*, 3, 4–70. doi:10.1177/109442810031002

[CIT0048] Vindbjerg E., Carlsson J., Mortensen E. L., Elklit A., & Makransky G. (2016). The latent structure of post-traumatic stress disorder among Arabic-speaking refugees receiving psychiatric treatment in Denmark. *British Medical Journal of Psychiatry*, 16, 309. doi:10.1186/s12888-016-0936-0 PMC5011808 27596249

[CIT0049] Wind T. R., Joshi P. C., Kleber R. J., & Komproe I. H. (2014). The effect of the postdisaster context on the assessment of individual mental health scores. *American Journal of Orthopsychiatry*, 84(2), 134–141. doi:10.1037/h0099385 24826929

[CIT0050] Zwart J. A., Dyb G., Hagen K., Ødegard K. J., Dahl A. A., Bovim G., & Stovner L. J. (2003). Depression and anxiety disorders associated with headache frequency. The Nord-Trøndelag Health Study. *European Journal of Neurology*, 10(2), 147–152. doi:10.1046/j.1468-1331.2003.00551.x 12603289

