# Corrigendum

**DOI:** 10.1080/20008198.2017.1342353

**Published:** 2017-06-21

The assessment of psychopathology among traumatized refugees: measurement invariance of the Harvard Trauma Questionnaire and the Hopkins Symptom Checklist-25 across five linguistic groups

Tim R. Wind^a^, Niels van der Aa^a^, Simone de la Rie^a^ and Jeroen Knipscheer^a,^ ^b^

^a^Department of Research, Arq Psychotrauma Expert Group, Diemen, the Netherlands; ^b^Department of Clinical Psychology, Utrecht University, Utrecht, the Netherlands

VOL. 8, 1321357

https://doi.org/10.1080/20008198.2017.1321357

The order of authors in this *European Journal of Psychotraumatology* article which is published online is incorrect. The correct order is provided below:

Tim R. Wind^a^, Niels van der Aa^a^, Jeroen Knipscheer^a,^ ^b^ and Simone de la Rie^a^

^a^Foundation Centrum ’45, Arq Psychotrauma Expert Group, Diemen, the Netherlands; ^a^Department of Clinical Psychology, Utrecht University, Utrecht, the Netherlands

