# Supplementary material for: The assessment of psychopathology among traumatized refugees: measurement invariance of the Harvard Trauma Questionnaire and the Hopkins Symptom Checklist-25 across five linguistic groups
Source: Eur J Psychotraumatol. 2017 Jun 9;8(sup2):1321357. doi: 10.1080/20008198.2017.1321357 (PMC5632793; doi:10.1080/20008198.2017.1321357)
Supplement: Supplementary material [file ZEPT_A_1321357_SM3628.docx]

Table S1

Unstandardized and standardized factor loadings of the 3-factor model of PTSD as estimated by the multigroup CFA representing configural invariance

|  | Indo-Iranian languages | | Niger-Congo languages | | Semitic languages | | South Slavic languages | | Germanic language | |
| --- | --- | --- | --- | --- | --- | --- | --- | --- | --- | --- |
|  | Unstand. | Stand. | Unstand. | Stand. | Unstand. | Stand. | Unstand. | Stand. | Unstand. | Stand. |
| *Factor 1* |  | |  | |  | |  | |  | |
| Item 1 | 1.26 | .78 | 1.90 | .88 | 1.28 | .79 | 1.35 | .80 | 1.68 | .86 |
| Item 2 | 1.20 | .77 | 1.40 | .82 | .94 | .69 | 1.15 | .76 | 1.60 | .85 |
| Item 3 | 1.15 | .76 | 1.22 | .77 | .90 | .67 | 1.07 | .73 | 1.55 | .84 |
| Item 8 | 1.05 | .73 | 1.23 | .78 | .91 | .67 | 1.93 | .89 | .93 | .68 |
| Item 16 | 1.16 | .76 | 1.02 | .71 | 1.19 | .77 | 1.08 | .73 | 1.56 | .84 |
| *Factor 2* |  | |  | |  | |  | |  | |
| Item 4 | .86 | .65 | 1.08 | .74 | .61 | .52 | .80 | .63 | .92 | .68 |
| Item 5 | .60 | .52 | .71 | .58 | .68 | .56 | .77 | .61 | .74 | .59 |
| Item 6 | 1.21 | .77 | 1.05 | .72 | 1.04 | .72 | 1.23 | .78 | 1.39 | .81 |
| Item 7 | .62 | .52 | .76 | .60 | .86 | .65 | 1.29 | .79 | 1.32 | .80 |
| Item 9 | .81 | .63 | 1.56 | .84 | .67 | .56 | .76 | .60 | 1.08 | .73 |
| Item 10 | 1.01 | .71 | .79 | .62 | .58 | .50 | .69 | .57 | .77 | .61 |
| Item 13 | 1.01 | .71 | .73 | .59 | .88 | .66 | 1.02 | .71 | 1.30 | .79 |
| Item 14 | 1.32 | .80 | .96 | .69 | .71 | .58 | .96 | .69 | 1.00 | .71 |
| *Factor 3* |  | |  | |  | |  | |  | |
| Item 11 | .94 | .69 | 2.05 | .90 | 1.03 | .72 | 1.31 | .80 | 2.61 | .93 |
| Item 12 | .28 | .27 | .29 | .28 | .12 | .12 | .36 | .34 | .66 | .55 |
| Item 15 | 1.49 | .83 | 1.03 | .72 | .83 | .64 | 1.45 | .82 | 1.83 | .88 |

Note. Unstand. = Unstandardized factor loadings; Stand. = Standardized factor loadings.

Table S2.

Thresholds of the PTSD-items of the HTQ as estimated by the multigroup CFA representing configural invariance

|  | Indo-Iranian languages | | | Niger-Congo languages | | | Semitic languages | | | South Slavic languages | | | Germanic language | | |
| --- | --- | --- | --- | --- | --- | --- | --- | --- | --- | --- | --- | --- | --- | --- | --- |
|  | T1 | T2 | T3 | T1 | T2 | T3 | T1 | T2 | T3 | T1 | T2 | T3 | T1 | T2 | T3 |
| Item 1 | -2.92 | -1.69 | -.45 | -4.30 | -2.21 | -.70 | -3.57 | -1.70 | -.42 | -3.15 | -1.72 | -.11 | -2.38 | -.54 | 1.39 |
| Item 2 | -1.75 | -.83 | .43 | -2.15 | -1.00 | .07 | -1.59 | -.77 | .25 | -2.13 | -.92 | .36 | -.97 | .56 | 2.16 |
| Item 3 | -2.30 | -1.30 | -.27 | -2.54 | -1.52 | -.42 | -2.18 | -1.14 | -.07 | -2.09 | -1.44 | .00 | -.63 | .69 | 2.06 |
| Item 4 | -1.62 | -.63 | .36 | -1.77 | -.50 | .59 | -1.67 | -.76 | .22 | -1.73 | -.67 | .58 | -1.98 | -.32 | 1.07 |
| Item 5 | -.58 | .17 | 1.05 | -.88 | .01 | .88 | -.96 | -.04 | .70 | -.93 | -.15 | .89 | -.78 | .35 | 1.49 |
| Item 6 | -2.19 | -1.23 | .05 | -1.80 | -.71 | .41 | -2.02 | -1.04 | .18 | -2.85 | -1.88 | .18 | -1.96 | -.33 | 1.38 |
| Item 7 | -1.88 | -1.06 | -.04 | -2.12 | -1.10 | .02 | -2.52 | -1.34 | -.20 | -3.52 | -1.71 | -.01 | -2.67 | -.93 | .89 |
| Item 8 | -2.24 | -1.68 | -.60 | -2.68 | -1.48 | -.58 | -3.33 | -2.07 | -.79 | -4.25 | -3.03 | -.80 | -1.40 | -.28 | .73 |
| Item 9 | -1.59 | -.74 | .19 | -2.76 | -1.07 | .38 | -1.69 | -.91 | .16 | -1.99 | -.99 | .34 | -1.87 | -.75 | .61 |
| Item 10 | -1.87 | -.76 | .52 | -1.31 | -.49 | .68 | -1.80 | -.86 | .16 | -1.73 | -.70 | .76 | -1.24 | .02 | 1.46 |
| Item 11 | -1.66 | -.80 | .29 | -3.25 | -1.92 | -.49 | -1.93 | -1.20 | .09 | -2.30 | -1.13 | .48 | -1.59 | .42 | 2.36 |
| Item 12 | -.09 | .41 | 1.19 | -.08 | .43 | 1.07 | -.14 | .28 | 1.02 | -.34 | .38 | 1.28 | -.32 | .79 | 1.71 |
| Item 13 | -1.59 | -.85 | .47 | -1.40 | -.24 | .69 | -2.03 | -.86 | .31 | -1.98 | -.98 | .52 | -1.66 | -.14 | 1.64 |
| Item 14 | -2.05 | -1.25 | -.13 | -2.34 | -1.14 | -.40 | -1.91 | -1.06 | -.17 | -2.15 | -1.15 | .20 | -1.08 | .04 | 1.20 |
| Item 15 | -2.49 | -1.18 | .12 | -1.92 | -1.00 | .17 | -1.81 | -1.00 | .17 | -2.51 | -1.02 | .71 | -1.65 | .18 | 2.09 |
| Item 16 | -2.70 | -1.27 | -.07 | -1.91 | -1.18 | -.05 | -2.74 | -1.70 | -.06 | -2.26 | -1.29 | .14 | -1.57 | .14 | 1.92 |

Note. T1 = first threshold; T2 = second threshold; T3 = third threshold.

Table S3.

Unstandardized and standardized factor loadings of the 2-factor model of anxiety and depression as estimated by the multigroup CFA representing configural invariance

|  | Indo-Iranian languages | | Niger-Congo languages | | Semitic languages | | South Slavic languages | |
| --- | --- | --- | --- | --- | --- | --- | --- | --- |
|  | Unstand. | Stand. | Unstand. | Stand. | Unstand. | Stand. | Unstand. | Stand. |
| *Factor 1* |  | |  | |  | |  | |
| Item 1 | 1.29 | .79 | .98 | .70 | 1.19 | .76 | .99 | .70 |
| Item 2 | 1.21 | .77 | 1.21 | .77 | 1.12 | .74 | 1.11 | .74 |
| Item 3 | .90 | .67 | .82 | .64 | .83 | .64 | .91 | .67 |
| Item 4 | 1.62 | .85 | .99 | .70 | 1.14 | .75 | 1.13 | .75 |
| Item 5 | 1.10 | .74 | .90 | .67 | .69 | .57 | .96 | .69 |
| Item 6 | .93 | .68 | .74 | .60 | .86 | .65 | .94 | .68 |
| Item 7 | .95 | .69 | 1.28 | .79 | .89 | .66 | 1.17 | .76 |
| Item 9 | 1.11 | .74 | 1.01 | .71 | 1.11 | .74 | 1.28 | .79 |
| Item 10 | 1.00 | .71 | .73 | .59 | .78 | .61 | .91 | .67 |
| *Factor 2* |  |  |  |  |  |  |  |  |
| Item 11 | .91 | .68 | .72 | .58 | .93 | .68 | .83 | .64 |
| Item 12 | .65 | .55 | .66 | .55 | .58 | .50 | .69 | .57 |
| Item 13 | .73 | .59 | .70 | .57 | .37 | .35 | .46 | .42 |
| Item 14 | .65 | .55 | .47 | .42 | .73 | .59 | .78 | .61 |
| Item 15 | .51 | .45 | .50 | .45 | .57 | .49 | .40 | .37 |
| Item 16 | .76 | .61 | 1.01 | .71 | .76 | .61 | .88 | .66 |
| Item 17 | 1.23 | .78 | 1.29 | .70 | 1.65 | .77 | 1.29 | .78 |
| Item 18 | 1.23 | .78 | 1.29 | .79 | 1.65 | .85 | 1.29 | .79 |
| Item 19 | .68 | .56 | .70 | .57 | .94 | .68 | 1.14 | .75 |
| Item 20 | .98 | .70 | .89 | .67 | .73 | .59 | .93 | .68 |
| Item 21 | 1.00 | .71 | 1.28 | .79 | 1.03 | .72 | 1.10 | .74 |
| Item 22 | .93 | .68 | 1.21 | .77 | .97 | .70 | .74 | .60 |
| Item 23 | .74 | .60 | .90 | .67 | .96 | .69 | 1.18 | .76 |
| Item 24 | 1.09 | .74 | 1.06 | .73 | .99 | .70 | 1.16 | .76 |
| Item 25 | 1.04 | .72 | .73 | .59 | .96 | .69 | 1.25 | .78 |

Note. Unstand. = Unstandardized factor loadings; Stand. = Standardized factor loadings.

Table S4.

Thresholds of the anxiety and depression items of the HSCL-25 as estimated by the multigroup CFA representing configural invariance

|  | Indo-Iranian languages | | | Niger-Congo languages | | | Semitic languages | | | South Slavic languages | | |
| --- | --- | --- | --- | --- | --- | --- | --- | --- | --- | --- | --- | --- |
|  | T1 | T2 | T3 | T1 | T2 | T3 | T1 | T2 | T3 | T1 | T2 | T3 |
| Item 1 | -1.78 | -.55 | .78 | -1.75 | -.90 | .51 | -1.98 | -.78 | .56 | -1.65 | -1.02 | .49 |
| Item 2 | -1.71 | -.54 | .54 | -2.61 | -1.35 | .18 | -1.94 | -.70 | .47 | -2.02 | -.99 | .46 |
| Item 3 | -1.52 | -.62 | .44 | -1.81 | -.47 | .68 | -1.99 | -.77 | .28 | -2.12 | -.93 | .60 |
| Item 4 | -2.41 | -1.12 | .20 | -1.52 | -.44 | .53 | -1.97 | -.86 | .39 | -2.95 | -1.59 | .17 |
| Item 5 | -1.31 | -.13 | .84 | -1.28 | -.49 | .70 | -1.50 | -.45 | .61 | -1.92 | -.58 | .78 |
| Item 6 | -1.19 | .06 | 1.12 | -.59 | .35 | 1.45 | -.89 | .16 | .97 | -1.44 | -.17 | 1.37 |
| Item 7 | -2.14 | -.88 | .40 | -2.10 | -.66 | .63 | -2.49 | -1.07 | .20 | -2.72 | -1.66 | .06 |
| Item 9 | -1.40 | -.38 | .71 | -1.49 | -.84 | .31 | -1.44 | -.30 | .83 | -1.81 | -.63 | .69 |
| Item 10 | -1.51 | -.65 | .54 | -1.31 | -.29 | .86 | -1.70 | -.90 | .16 | -1.77 | -.95 | .50 |
| Item 11 | -1.88 | -.82 | .29 | -2.04 | -.34 | .85 | -2.24 | -1.03 | .20 | -2.06 | -.86 | .42 |
| Item 12 | -.89 | -.22 | .56 | -1.03 | -.39 | .36 | -1.06 | -.40 | .47 | -1.20 | -.25 | .77 |
| Item 13 | -1.09 | -.22 | .56 | -1.20 | -.53 | .39 | -.95 | -.20 | .37 | -1.13 | -.39 | .32 |
| Item 14 | -.92 | .00 | .57 | -.89 | -.25 | .39 | -.97 | -.25 | .51 | -1.36 | -.59 | .29 |
| Item 15 | -.68 | .12 | 1.06 | -1.30 | -.36 | .69 | -1.06 | -.14 | .84 | -.52 | .39 | 1.29 |
| Item 16 | -2.12 | -1.35 | -.44 | -2.62 | -1.45 | -.68 | -2.71 | -1.85 | -.64 | -2.47 | -1.65 | -.59 |
| Item 17 | -2.07 | -1.08 | -.24 | -2.44 | -1.42 | -.51 | -2.37 | -1.52 | -.19 | -2.43 | -1.35 | .08 |
| Item 18 | -2.59 | -1.53 | -.17 | -2.68 | -1.31 | .05 | -3.48 | -1.85 | -.19 | -3.01 | -1.49 | .07 |
| Item 19 | -1.74 | -.89 | -.06 | -2.02 | -1.01 | -.06 | -2.06 | -1.06 | -.18 | -1.82 | -.80 | .63 |
| Item 20 | .03 | .81 | 1.51 | -.60 | .28 | .99 | -.06 | .58 | 1.39 | -.16 | .95 | 1.71 |
| Item 21 | -1.13 | -.42 | .68 | -1.76 | -.87 | .27 | -1.22 | -.48 | .30 | -1.88 | -.57 | .92 |
| Item 22 | -2.62 | -1.71 | -.40 | -2.90 | -1.71 | -.05 | -3.16 | -1.77 | -.56 | -2.03 | -1.03 | .04 |
| Item 23 | -1.23 | -.35 | .59 | -1.51 | -.51 | .89 | -1.79 | -.81 | .36 | -1.38 | -.33 | 1.01 |
| Item 24 | -1.81 | -.68 | .53 | -1.82 | -.56 | .63 | -2.42 | -1.08 | .19 | -1.88 | -.63 | .88 |
| Item 25 | -1.31 | -.44 | .52 | -1.26 | -.37 | .45 | -1.13 | -.50 | .34 | -1.60 | -.57 | .74 |

Note. T1 = first threshold; T2 = second threshold; T3 = third threshold.
